# Volume-controlled inverse ratio ventilation improves safe apnea time in obese patients during the induction of general anesthesia: a randomized controlled trial

**DOI:** 10.3389/fmed.2025.1574634

**Published:** 2025-05-01

**Authors:** Yonghai Zhang, Bin Li, Chang Xu, Yan Wu, Ling Ma, Fan Yang, Hanxiang Ma, Xinli Ni

**Affiliations:** ^1^Department of Anesthesiology and Perioperative Medicine, General Hospital of Ningxia Medical University, Yinchuan, China; ^2^Department of Anesthesiology, People’s Hospital of Ningxia Hui Autonomous Region, Yinchuan, China

**Keywords:** safe apnea time, pre-oxygenation, inverse ratio ventilation, functional residual capacity, obesity, oxygen saturation, expiratory oxygen fraction

## Abstract

**Background:**

Inverse ratio ventilation theoretically increases oxygenation in obese patients. However, it is unknown whether the use of inverse ratio ventilation prolongs the safe apnea time during the induction of anesthesia. The primary objective of our study was to compare the safe apnea time between obese surgical patients receiving inverse ratio ventilation and conventional ratio ventilation during the induction of anesthesia.

**Methods:**

This study is a prospective, randomized controlled trial. Forty obese patients who underwent elective operation under general anesthesia with endotracheal intubation were randomly allocated into the conventional ratio ventilation (CRV) group (*n* = 20) and inverse ratio ventilation (IRV) group (*n* = 20). After the patients were preoxygenated through a face mask for 3 min, anesthesia induction was performed. When the patients lost consciousness and spontaneous breathing disappeared, non-invasive positive pressure ventilation was performed for 5 min, and the inspiratory-to-expiratory (I:E) ratio was set as 1:2 in the CRV group and 2:1 in the IRV group. Heart rate, systolic blood pressure, diastolic blood pressure, and pulse oxygen saturation were recorded at four time points: (i) before pre-oxygenation (T_0_), (ii) pre-oxygenation for 3 min (T_1_), (iii) non-invasive positive pressure ventilation for 3 min (T_2_), and (iv) non-invasive positive pressure ventilation for 5 min (T_3_). Arterial blood was collected at T_0_, T_1_, and T_3_ for arterial blood gas analysis, and arterial oxygen partial pressure and carbon dioxide partial pressure were recorded. The patient’s expiratory oxygen fraction at T_1_, T_2_, and T_3_ were recorded. Peak airway pressure, plateau pressure and mean airway pressure were record at T_2_ and T_3_. The safe apnea time was recorded in both groups.

**Results:**

Forty patients completed the study. Baseline parameters were comparable between the two groups. Safe apnea time was significantly longer (210.40 ± 47.47 vs. 153.80 ± 41.54 s, mean difference [95% CI], 56.55 [28.00–85.10], *p* = 0.0003) and the expired O_2_ fraction was higher (87.60 ± 2.39 vs. 91.60 ± 1.79, mean difference [95% CI], 4.00 [2.65–5.35], *p* < 0.0001) at T_3_ in the IRV group compared to the CRV group.

**Conclusion:**

Volume-controlled inverse ratio ventilation at an I:E ratio of 2:1, compared to conventional ratio ventilation, provided a longer safe apnea time and higher expired O_2_ fraction in obese patients during the induction of anesthesia.

## 1 Introduction

Obesity has become one of the major public health problems Its prevalence is alarming (1) and it is associated with numerous comorbidities when obese patients undergo general anesthesia, which include obesity hypoventilation, difficult intubation, obstructive sleep apnea, and other comorbidities (2, 3). Therefore, this raises many concerns and challenges for anesthesiologists. Obese patients are prone to hypoxemia during the induction of general anesthesia due to their altered respiratory mechanics and physiology and changes in respiratory function after anesthesia (4). Their safe apnea time is generally less than 3 min (5, 6), which is significantly shorter than the 8.9 min in healthy people (7). Furthermore, data suggests that there is a negative linear correlation between safe apnea time and increasing obesity (6). The shorter safe apnea time may be related to the decreased functional residual capacity (FRC) and increased oxygen consumption in obese patients (8). Several measures, including head-up (9) or beach-chair positioning (10), non-invasive ventilatory support (5), and high-flow nasal oxygen (11) can improve safe apnea time to varying degrees at present.

In some clinical cases, the anesthesiologist may be required to complete the induction of general anesthesia in an obese patient alone, and both hands have to be used to control the obese patient’s airway. At this time, using mechanical ventilation via mask is feasible and effective to provide respiratory support after the breath disappears. Inverse ratio ventilation (IRV) typically increases oxygenation by increasing the mean airway pressure (MAP), which happens due to more time spent on the higher pressure portion of the cycle (12). Increased MAP can elevate mean alveolar pressure and thus transpulmonary pressure, which can prevent the reduction of FRC, improve alveolar ventilation/blood flow ratio, and finally improve the patient’s oxygenation status (13). Studies have evaluated the effect of IRV in improving oxygenation and gas exchange in obese adult patients (14) and obese children (15) undergoing laparoscopic surgery under general anesthesia and it was demonstrated that IRV can improve oxygenation status significantly compared to the control groups without adverse respiratory and hemodynamic effects. However, it is unclear whether IRV can improve safe apnea time in obese patients during induction of general anesthesia.

We hypothesized that volume-controlled inverse ratio ventilation with an I:E ratio of 2:1 for pre-oxygenation after the breath disappears would result in longer safe apnea time than conventional ratio ventilation with an I:E ratio of 1:2 in obese patients during induction of general anesthesia.

## 2 Methods

### 2.1 Study design and randomization

This randomized, double-blind, controlled study was approved by the Institutional Ethics Committee of the General Hospital of Ningxia Medical University (2020-718) in August 2020 and was also conducted at General Hospital of Ningxia Medical University. The study was also registered at ClinicalTrials.gov (NCT04627883) in August 2020. Written informed consent was obtained from all subjects participating in the trial before the surgery. This study was reported in accordance with the CONSORT statement. Forty-eight patients with American Society of Anesthesiologists (ASA) physical status II–III, aged between 18 and 65 years, a body mass index (BMI) ≥ 28 kg · m^−2^ (Chinese standards for diagnosis of obesity), and a clinical need for endotracheal intubation who were scheduled for elective surgery under general anesthesia were enrolled in this study from August 17, 2020 to January 1, 2022. The exclusion criteria included requiring rapid sequence induction, anticipated or history of difficult airway, uncontrolled gastric reflux disease, moderate to severe comorbidity (respiratory or renal disease, ischemic heart or uncontrolled hypertension or pulmonary hypertension disease, cerebrovascular disease), abnormal basal metabolic rate, and abnormal hemoglobin or hematocrit.

Before patient recruitment, group randomization was performed using an online web-based randomization tool^1^ with block sizes of six and an allocation ratio of 1:1 by an investigator not involved in the study. Forty-eight eligible participants who underwent elective operation under general anesthesia with endotracheal intubation were randomly allocated into conventional ratio ventilation group (CRV group, *n* = 24) and inverse ratio ventilation group (IRV group, *n* = 24) based on the allocation sequence. An anesthetist prepared concealed group assignments in consecutively numbered, sealed, opaque envelopes together with a detailed information of the study ventilation preparation, and then sets ventilation parameters to be adjusted and masked them by a piece of paper. Thereafter, he was no longer involved in the study. All study parameters were observed by another investigator. The randomization was not disclosed to any of the personnel who recorded the study parameters or the patient throughout the study.

### 2.2 Preparation and anesthesia

All the patients were instructed to fast for at least 8 h and no premedication drugs were given before the induction of anesthesia. After entering the operation room, intravenous access was established in all the patients and they were monitored with an electrocardiogram, non-invasive blood pressure, and pulse oximeter at 20° head-up inclination position. Radial artery was punctured and cannulated under local anesthesia for collecting the arterial blood gases. The inspired O_2_ concentration was set at 100% and the breathing circuit system was flushed with the O_2_ bypass for 30 s before being connected to the patients. Pre-oxygenation was performed through a well-fitted facemask firmly applied by four headbands and connected to the anesthesia machine (Fabius GS; Dräger Medical SAS, Antony, France) delivering 10 L · min^−1^ of 100% oxygen. Both groups adopted the volume-controlled ventilation mode. The ventilator parameters were set as follows: RR represents respiration rate, 12 times · min^−1^, tidal volume, 8 mL × ideal body weight (IBW), IBW = [50 (men)/45.5 (women) + 0.91 × (height in cm − 152.4 cm)], and the I:E ratio was set 1:2 in CRV group and 2:1 in the IRV group. After pre-oxygenation (breath normally) for 3 min, general anesthesia was induced using midazolam (0.05–0.1 mg · kg^−1^), propofol (1–2 mg · kg^−1^), sufentanil (0.3–0.5 ug · kg^−1^), and rocuronium (0.9 mg · kg^−1^). A nasopharyngeal airway (the nasal cavity was inducted with topical anesthesia and lubricated before anesthesia) was inserted in all patients, and jaw-thrust was applied when the patient lost consciousness and spontaneous breathing disappeared to ensure patent airway, and mechanical ventilation was initiated via facemask based on the pre-designed ventilation patterns immediately. After 5 min of ventilation, facemask was removed, and tracheal intubation was performed by using a video laryngoscope. Successful intubation was confirmed with capnography (to ventilate approximately 300 mL by using breathing bag). Removing the breathing tube and keeping it disconnected, the safe apnea time, which was defined as the time required for SpO_2_ to desaturate to 93% measured after the facemask was removed, was recorded, and ventilation was performed after the breathing tube was connected to restore oxygen saturation immediately.

### 2.3 Data collection

The primary outcome of the study was the safe apnea time in seconds measured after removal of the facemask from patients to the decrease of SpO_2_ to 93%. The following data were also recorded and analyzed as secondary endpoints: Expired O_2_ fraction (FeO_2_) at preoxygenation for 3 min (T_1_), non-invasive positive pressure ventilation for 3 min (T_2_) and 5 min (T_3_); PaO_2_ and PaCO_2_ at before preoxygenation(T_0_), T_1_ and T_3_; Ppeak, Pplat, and MAP (MAP = 0.5 (PIP – PEEP) × (Ti/T_tot_) + PEEP) at T_2_ and T_3_; heart rate (HR), systolic blood pressure (SBP) and diastolic blood pressure (DBP) at T_0_, T_1_, T_2_, and T_3_.

### 2.4 Statistical analysis

Sample size estimation was performed using NCSSPASS software (version 11.0.7, update time January 22, 2013). As there was no similar study published, a pilot study was conducted in 12 patients to calculate the sample size. Considering the safe apnea time as the primary outcome in the CRV group (134 ± 37 s) and IRV group (195 ± 16 s) with a confidence interval of 95 and 99%, the minimum required sample size to obtain statistically significant result was calculated to be 20 patients in each group. Finally, we recruited 48 new patients who were equally allocated to the CRV and IRV groups.

Statistical analysis was performed by SPSS (IBM SPSS Statistics, Version 25, IBM Germany, Ehningen, Germany). Data are presented as the mean ± standard deviation (SD) or median (interquartile range, IQR) as required or numbers (*n*). The normality of quantitative data was tested using the Kolmogorov and Smirnov test. The equality of variance was tested by the Levene test if required. For intergroup comparisons of the parametric continuous variables, Student’s *t*-test was used. Continuous variables that did not meet the criteria for parametric testing were evaluated using the Mann–Whitney U test. Repeated-measures data were analyzed by repeated-measures analysis of variance. To verify whether categorical variables differed significantly between the groups, the chi-square test was used for comparison. A *p* value < 0.05 was considered to be statistically significant in a two-sided test.

## 3 Results

### 3.1 Flow diagram and patient characteristics

**Figure 1:**
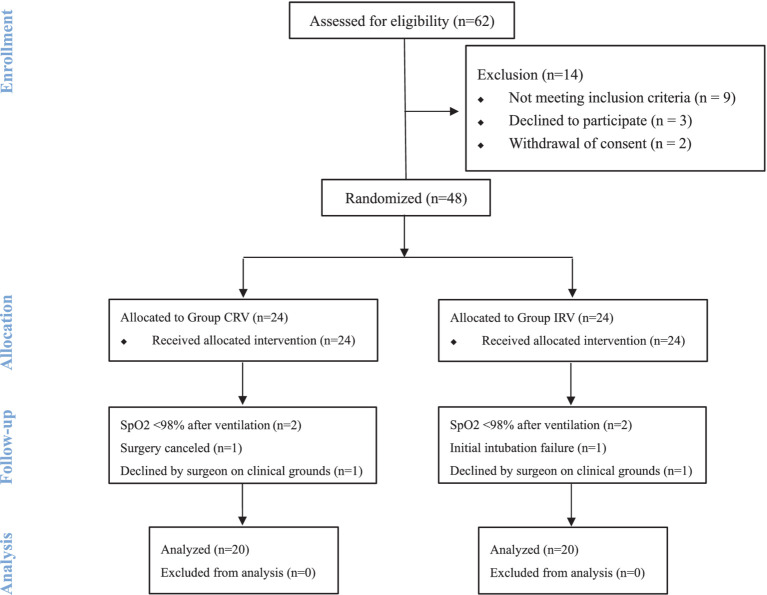
Enrolment and randomization of the patients.

The CONSORT study flow diagram is presented in Figure 1. Enrolment took place between August 17, 2020, and January 1, 2022. This prospective, randomized, and controlled study originally considered 62 eligible patients, of whom 48 were enrolled and randomly assigned to the CRV group (*n* = 24) and IRV group (*n* = 24). There was no statistically significant difference between the four groups with regard to age, sex, BMI, hemoglobin (Hb), and ASA physical status, as shown in Table 1.

**Table 1:** Patient characteristics.

Variables	CRV group	IRV group	*P* value
Age, years	48.35 ± 10.26	45.85 ± 10.21	0.4446
Sex, male/female	13/7	10/10	0.5231
BMI, kg · m^−2^	31.15 ± 3.00	31.20 ± 1.67	0.9484
Hb, g · L^−1^	132.50 ± 9.90	126.90 ± 7.61	0.0520
ASA, II/III	19/1	18/2	0.9999

CRV, conventional ratio ventilation; IRV, inverse ratio ventilation; BMI, body mass index; Hb, hemoglobin; ASA, American Society of Anesthesiologists. Data are presented as the mean ± SD or numbers (*n*).

### 3.2 Safe apnea time

**Figure 2:**
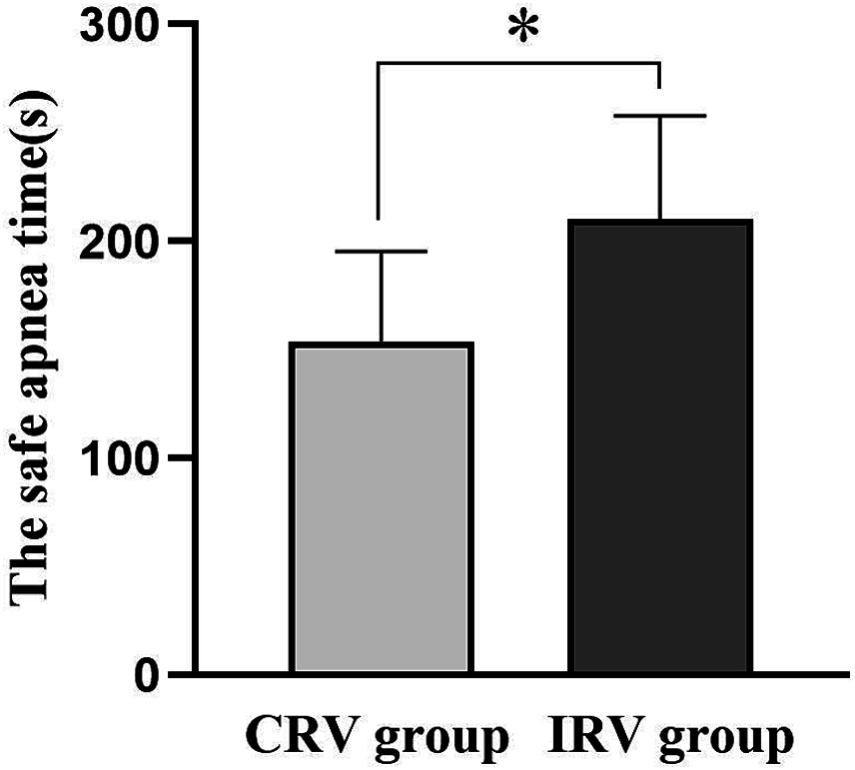
The safe apnea time in both groups. CRV, conventional ratio ventilation; IRV, inverse ratio ventilation. Data are presented as the mean ± SD. *p < 0.05 compared with the CRV group.

For the primary outcome, the safe apnea time for patients in the CRV group was 153.80 ± 41.54 s, and that in the IRV group was 210.40 ± 47.47 s. The mean difference [95% CI] between them was 56.55 [28.00–85.10]. Compared with the CRV group, the safe apnea time in the IRV group was longer, and the difference was statistically significant (*p* = 0.0003), as shown in Figure 2.

### 3.3 Secondary endpoints

**Figure 3:**
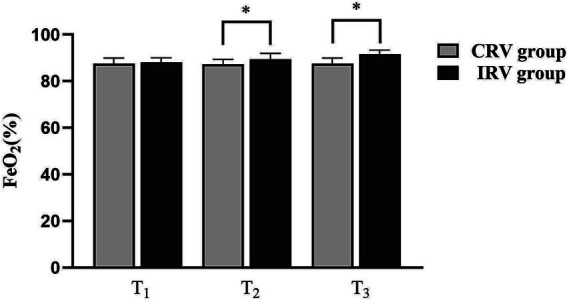
Changes of FeO_2_ in both groups. CRV, conventional ratio ventilation; IRV, inverse ratio ventilation. Data are presented as the median (IQR). *p < 0.05 compared with the CRV Group.

As shown in Figure 3, FeO_2_ was comparable in both the groups at T_0_. However, at T_2_ and T_3_, IRV group (89.45 ± 2.54, 91.60 ± 1.79) had significantly higher FeO_2_ as compared to CRV group (87.20 ± 2.17, 87.60 ± 2.39) (*p* = 0.0046, *p* < 0.0001).

According to the results of arterial blood gas analysis, the indexes were compared between the two groups. There was no statistically significant difference in PaO₂ and PaCO_2_ between the two groups of patients at T_0_, T_1_, and T_3_ (*p* > 0.05). Compared to T_0_, the PaO₂ of the two groups of patients increased significantly at T_1_ and T_3_, and the PaCO_2_ of the two groups of patients increased at T_3_ and only increased at T_1_ in the CRV group, and the differences were statistically significant (*p* < 0.05), as shown in Table 2.

**Table 2:** PaO_2_ and PaCO_2_ in the two groups.

Variables	Time points	CRV group	IRV group	*P* value
PaO₂, mmHg	T_0_	72.67 ± 4.91	75.85 ± 6.88	0.1009
	T_1_	351.70 ± 44.23*	374.30 ± 57.59*	0.1716
	T_3_	359.00 ± 44.85*	381.20 ± 48.46*	0.1409
PaCO_2_,mmHg	T_0_	35.99 ± 2.58	34.96 ± 2.82	0.2351
	T_1_	38.12 ± 5.58*	36.08 ± 3.59	0.1774
	T_3_	43.05 ± 5.85*	41.99 ± 4.54*	0.5261

CRV, conventional ratio ventilation; IRV, inverse ratio ventilation; PaO_2_, partial pressure of arterial oxygen; PaCO_2_, partial pressure of arterial carbon dioxide; OI, oxygenation index. Data are presented as the mean ± SD. *P < 0.05 compared with the T_0_.

Compared with the CRV group, the Ppeak and Pplat of the IRV group decreased at both T_2_ and T_3_, and the differences were statistically significant (all *p <* 0.05), as shown in Table 3. MAP was significantly higher in the IRV group compared with the CRV group at both T_2_ and T_3_ (*p* < 0.0001), as shown in Table 3.

**Table 3:** Ppeak, Pplat, and MAP in the two groups.

Variables	Time points	CRV group	IRV group	*P* value
Ppeak, cmH_2_O	T_2_	13.30 ± 1.26	12.50 ± 0.95	0.0290
	T_3_	13.35 ± 1.04	12.30 ± 0.86	0.0013
Pplat, cmH_2_O	T_2_	12.35 ± 1.04	11.40 ± 0.94	0.0044
	T_3_	12.60 ± 0.99	11.60 ± 0.88	0.0018
MAP, cmH_2_O	T_2_	2.20 ± 0.21	4.46 ± 0.42	<0.0001
	T_3_	2.20 ± 0.17	4.47 ± 0.35	<0.0001

CRV, conventional ratio ventilation; IRV, inverse ratio ventilation; Ppeak, Peak pressure; Pplat, plateau pressure; MAP, mean airway pressure. Data are presented as the mean ± SD.

As shown in Table 4, there was no statistically significant difference in the HR, systolic blood pressure (SBP), and diastolic blood pressure (DBP) between the two groups of patients at T_0_, T_1_, T_2_, and T_3_ (*p* > 0.05). Compared with T_0_, the HR of patients in the two groups decreased at T_1_, T_2_, and T_3_, the SBP and DBP decreased at T_2_ and T_3_, and the difference was statistically significant (*p* < 0.05).

**Table 4:** Changes of HR, SBP, and DBP in the two groups.

Variables	Time points	CRV group	IRV group	*P* value
HR, beat · min^−1^	T_0_	74.65 ± 10.36	74.85 ± 6.44	0.9420
	T_1_	65.95 ± 10.11*	70.20 ± 6.90*	0.1287
	T_2_	60.25 ± 7.27*	61.90 ± 5.82*	0.4330
	T_3_	58.80 ± 5.39*	61.70 ± 4.98*	0.0849
SBP, mmHg	T_0_	124.80 ± 19.50	133.30 ± 12.13	0.1081
	T_1_	118.00 ± 12.52	125.10 ± 11.89	0.0738
	T_2_	113.80 ± 10.17*	119.50 ± 10.83*	0.0971
	T_3_	110.80 ± 12.87*	114.50 ± 13.40*	0.3789
DBP, mmHg	T_0_	65.45 ± 8.80	66.30 ± 8.20	0.7537
	T_1_	62.85 ± 7.72	64.35 ± 7.87	0.5465
	T_2_	58.70 ± 5.79*	62.05 ± 6.34*	0.0889
	T_3_	58.45 ± 5.07*	61.85 ± 6.69*	0.0781

CRV, conventional ratio ventilation; IRV, inverse ratio ventilation; HR, heart rate; SBP, systolic blood pressure; DBP, diastolic blood pressure. Data are presented as the mean ± SD. *P < 0.05 compared with the T_0_.

## 4 Discussion

The main finding of our study was that volume-controlled inverse ratio ventilation with an I:E ratio of 2:1 prolonged safe apnea time by 57 s and significantly increased FeO_2_ levels in the obese surgical patients compared with conventional proportional ventilation with an I:E ratio of 1:2 during induction of general anesthesia. In addition, compared with conventional ventilation, inverse ratio ventilation with a 2:1of I:E ratio did not result in carbon dioxide retention or hemodynamic changes.

The main purpose of pre-oxygenation during the induction period of general anesthesia is to increase the oxygen/nitrogen ratio in the lung resting volume (functional residual capacity), so as to prolong the safe apnea time when breathing disappears and maintain SpO_2_ within a safe range. Clinically, obese patients have a significantly shorter safe apnea time than normal patients and are more likely to lapse into hypoxemia (6, 16). Even if obese patients are pre-oxygenated for 5 min, the SpO_2_ of patients may still drop rapidly to 90% within 2–3 min after induction without ventilation (5, 6).

This phenomenon is mainly related to the disorder of ventilation/perfusion ratio and the reduction of FRC in obese patients, and the inhalation of 100% oxygen during the anesthesia induction period. In obese patients, the redistribution of ventilation toward the lung apices with the bulk of perfusion delivered to the bases has been observed during spontaneous breathing at the end of exhalation, particularly in patients with a reduction in expiratory reserve of less than 300 mL (17). On the other hand, the cephalic displacement of the diaphragm by the abdominal fat in obese patients affects the lung capacity, producing a restrictive ventilatory disorder whose hallmark is the reduction in the FRC and decrease in the expiratory reserve volume (18). Furthermore, Jones RL et al. shown that with an increase in body mass index (between 25 and 35), the decreases in FRC and expiratory reserve volumes are more significant (19). In addition, inhalation of highly concentrated oxygen can lead the formation of reabsorption atelectasis during the intubation time, which will further reduce FRC (20). Finally, with the reduction of oxygen storage in the lungs, obese patients are more likely to develop hypoxemia during induction of general anesthesia.

At present, there are several methods to improve the safe apnea time in obese patients without ventilation, such as choosing an appropriate body position like head-up (9) or beach-chair positioning (10), non-invasive positive pressure ventilatory support (5), high-flow nasal oxygen (11), and selecting an appropriate oxygen concentration during pre-oxygenation (20). The objective of these methods is to increase the lung oxygen reserve of the patients by reducing atelectasis and improving lung volume (2). A study of induction positions in obese patients found that pre-oxygenation in the head-up position prolonged the safe apnea time by an average of 50–60 s compared with the supine position (9). A previous study also has found that the use of high-flow nasal oxygen techniques during intubation can extend the safe apnea time by more than 100 s (11). The combined use of CPAP Continuous Positive Airway Pressure and PEEP Positive End-Expiratory Pressure during induction in morbidly obese patients can extend the safe apnea time by more than 1 min (5). Compared with the above methods, inverse ratio ventilation can also be used as an effective and feasible method to prolong the safe apnea time in obese patients.

In the clinic, during mechanical ventilation under general anesthesia, the I:E ratio is usually set at 1:2 or 1:1.5 to approximate the normal inspiratory and expiratory physiology. However, sometimes we need to use the inverse ratio ventilation to improve oxygenation in patients with reduced FRC, such as neonates and ARDS Acute Respiratory Distress Syndrome patients (21, 22). In our study, we found that the I:E ratio 2:1 of volume-controlled ventilation not only prolongs safe apnea time by an average of 57 s but also increases FeO_2_ in obese surgical patients during the induction of general anesthesia. The main reason for this is that inverse ratio ventilation increases the mean airway pressure and dynamic compliance by lengthening the inspiratory time and shortening expiratory time, which avoids alveolar collapse and lung atelectasis, increases oxygen reserve, and improves the patient’s oxygenation status (12). Jo et al. shown that inverse ratio ventilation can improve intraoperative arterial oxygenation in morbidly obese patients undergoing bariatric surgery, which may be related to the fact that it can cause an increase in mean airway pressure and dynamic compliance, and finally improve alveolar collapse (23). In addition, intrinsic PEEP produced due to the relatively shortened expiration time of inverse ratio ventilation also helps to improve the oxygen saturation of the patient (24). In clinical practice, FeO_2_ is usually used to measure the effect of pre-oxygenation, and the ideal endpoint of pre-oxygenation and denitrification is defined as FeO_2_ of 90% or expired N_2_ concentration of 5%. Our research results suggest that the use of inverse ratio ventilation can improve FeO_2_ at a ventilation for 3 and 5 min, and this may also be the objective reason why inverse ratio ventilation prolongs safe apnea time.

We compared the PaO_2_ of the two groups at different time points and found that there was no statistical difference. Several studies found that the oxygen reserve mainly in the respiratory system, and not in the arterial blood, when inhaling air instead of inhaling 100% O_2_ (25, 26). Jense et al. found that the safe apnea time was negatively correlated with BMI, but without any correlation with the PaO_2_ in obese patients (6). In the Mir et al. study, it was also found that compared with mask pre-oxygenation, the rapid nasal oxygen inhalation can prolong the safe apnea time in patients undergoing emergency surgery, but there was no difference in PaO_2_ between the two groups (27). Our results are consistent with all these studies.

We also fund I:E ratio 2:1 volume-controlled inverse ratio ventilation did not cause carbon dioxide retention or hemodynamic changes. Consistent with our research results, Youn Yi Jo et al. found that there was no significant difference in PaCO_2_ between different I:E ratios in obese patients undergoing bariatric surgery, and inverse ratio ventilation had no effect on CO_2_ excretion (23). Previous studies found that patients did not experience serious adverse cardiovascular events when the I:E ratio was chosen to be 2:1 (28). This is also the basis for our study to choose 2:1 as the I:E ratio for non-invasive ventilation.

There are also some limitations of our study. First, this is a single-center study, and it is necessary to expand the sample in future research to further clarify the effect of volume-controlled inverse ratio ventilation on the safe apnea time in obese patients. Second, all patients were not ventilated for several minutes after the induction of anesthesia, posing a risk of lung injury. Third, we only used PaCO_2_ to observe whether inverse ratio ventilation would lead to carbon dioxide retention, but neglected to record the changes in end-tidal carbon dioxide, which may be more convenient and valuable. Fourth, we only chose the volume-controlled inverse ratio ventilation mode, and further research is needed on the effect of pressure-controlled inverse ratio ventilation mode on the safe apnea time in obese patients. Fifth, although we did not monitor gastric distention caused by non-invasive positive pressure ventilation, the Ppeak was well below the opening pressure of the upper esophageal sphincter (29), which may indicate that patients are unlikely to experience gastric distension.

In summary, volume-controlled inverse ratio ventilation of I:E ratio 2:1 in obese patients during the anesthesia induction resulted in longer safe apnea time and higher expired O_2_ fraction without carbon dioxide retention or hemodynamic changes compared to conventional ratio ventilation, which should be considered during the induction of anesthesia in obese surgical patients.

## Data Availability

The raw data supporting the conclusions of this article will be made available by the authors, without undue reservation.

## Footnotes

^1^ http://www.randomizer.org/
